# Impact of Vulvodynia on the Quality of Life of Women: A Rapid Review

**DOI:** 10.3390/jcm15010070

**Published:** 2025-12-22

**Authors:** María Fernanda Callirgos Escajadillo, Marina Gómez de Quero Córdoba, Marta Garrigues-Ramón, Sagrario Gómez-Cantarino, Adolfo Romero-Arana, Elena Arroyo-Bello

**Affiliations:** 1 La Paz University Hospital, Madrid Health Service, 28046 Madrid, Spain; mfcallirgos99@gmail.com; 2 Grupo de Investigación CARING-161, Universitat Rovira i Virgili, 43003 Tarragona, Spain; marina.gomezdequero@urv.cat; 3 Fundación Jiménez Díaz School of Nursing, Health Research Institute, Fundación Jiménez Díaz University Hospital, Universidad Autónoma de Madrid (IIS-FJD, UAM), 28040 Madrid, Spain; marta.garrigues@quironsalud.es (M.G.-R.); elena.arroyo@quironsalud.es (E.A.-B.); 4 Faculty of Physiotherapy and Nursing, Toledo Campus, Avda Carlos III, 45071 Toledo, Spain; sagrario.gomez@uclm.es

**Keywords:** vulvodynia, quality of life, women’s health, chronic pain, sexual function, multidisciplinary care

## Abstract

**Background:** Vulvodynia is a chronic vulvar pain syndrome with multifactorial etiology and unclear pathophysiology. Despite its high prevalence, it remains underdiagnosed and under-researched, with significant repercussions for women’s physical, psychological, and social well-being. **Objective:** To synthesize the available scientific evidence on quality of life (QoL) in women diagnosed with vulvodynia, identifying the main affected domains and the assessment tools used in the literature. **Methods:** A rapid review was conducted following PRISMA 2020 and Cochrane Rapid Reviews guidelines. Searches were performed in PubMed, Web of Science, Scopus, CINAHL, Biblioteca Virtual en Salud, and CUIDEN without date or geographic restrictions. Studies including adult women diagnosed with vulvodynia and reporting QoL outcomes were eligible. Data was extracted and synthesized narratively, and methodological quality was assessed using the Mixed Methods Appraisal Tool (MMAT). **Results:** Twenty studies published between 2006 and 2025 met the inclusion criteria (13 quantitative and 7 qualitative). Vulvodynia was consistently associated with reduced QoL across physical, psychological, and social dimensions. The most frequently reported issues were chronic vulvar pain, sexual dysfunction, anxiety, depression, and social withdrawal. Tools such as SF-12, SF-36, WHOQOL-BREF, DLQI, Skindex-29, and SQLQ-F were commonly used, although heterogeneity among instruments limited comparability. Multidisciplinary interventions combining physiotherapy and psychological therapy showed improvements in emotional and physical well-being, though sexual dysfunction often persisted. **Conclusions:** Vulvodynia substantially impairs women’s quality of life, reflecting complex biopsychosocial interactions. The findings highlight the need for standardized QoL measures and gender-sensitive, multidisciplinary approaches to diagnosis, management, and research.

## 1. Introduction

Vulvodynia is defined as a vulvar syndrome characterized by pain, burning, stinging, and sensory dysfunctions such as allodynia and/or hyperesthesia [1,2,3]. It is a chronic entity that, despite not having an attributable organic cause, combines pathophysiological factors from various spheres, such as inflammatory, immunomodulatory, infectious and psychological mechanisms [1,2].

Epidemiologically, the prevalence of vulvodynia is growing and is estimated to be between 10% and 28% of women worldwide, especially among young women of reproductive age [4,5]. Despite these findings, less than half of patients are estimated to seek medical attention, which reflects a significant degree of underdiagnosis and a lack of reliable epidemiological data, particularly in developing countries [3,4,5].

According to the scientific literature, vulvodynia can be classified into a more common or predominant form known as localized provoked vulvodynia, in which the signs and symptoms are produced by direct superficial or deep contact [1,2,3,6], and various subtypes among those that can be found are those triggered by allergic reactions or hormonal imbalances [1]. However, direct contact is not inherent to the clinic, since in the absence of stimulus, symptoms can appear suddenly or spontaneously, without the need for prior contact of an exogenous agent with the external or internal vulvar mucosa [1,2,3,4,6].

Vulvodynia has a significant negative effect on the sexual health of women, leading to the development of dyspareunia and vulvar hypersensitivity, which can affect self-esteem and alter libido and partner relationship, leading to long-term deterioration of quality of life and mental health, reflecting the need for a comprehensive approach [7,8].

To date, no fixed etiology has been identified for its onset and chronicity or as a clear risk factor. It is considered a disease with a large multifactorial component, on which multiple triggers can be identified, such as chronic infections, hormonal imbalances, hypersensitivity reactions, environmental and hygienic factors, genetic alterations, muscle-nerve hyperresponsiveness or even a set and synergy of several of these factors [3,4,6]. Academic recognition of this multifactorial etiology was consolidated in 2015, marking a turning point in the understanding of the complex origins of the disease. The limited social and professional awareness of vulvodynia, together with the limited training in its diagnosis and approach, underscores the need to intensify clinical and epidemiological research [3].

The interaction of these factors with psychological and psychosocial components tends to aggravate the clinical course and make diagnosis difficult [3,4,6]. It has been proposed that educating and using cognitive behavioral therapy may be more effective than topical pharmacology or physical therapy [7,8]. However, the conceptualization of pain still tends toward a dualistic view, considering it mainly as a medical or psychological problem. Therefore, current treatments are focused mainly on pharmacotherapy, surgery, or psychotherapy, although with low success rates and without optimal therapies [5].

The pathophysiology of vulvodynia is also unclear; pain can originate from an imbalance in the tonicity of the pelvic musculature, generating a proliferation of anomalous nerve fibers and excess reflex innervation as an excessive reaction [1,4,9]. Factors such as neuroimmunomodulatory mechanisms that induce inflammation, increased tension in myofascial tissues, and even pathological increases in sensory receptors can potentiate symptoms [1,3,9]. A psychological component, undervalued and complex, tends to aggravate the course and correct diagnosis of the disease [3,4,6]. Current evidence highlights that couple therapy, structured partner involvement, and the contribution of sexual therapists play a significant role in improving relational adjustment, reducing pain-related distress, and enhancing sexual and emotional quality of life in women with vulvodynia [3,5,7,9].

Its correct diagnosis poses an important challenge for public health, since it is very commonly confused with other vulvar pathologies [3,4,6]. There is also no curative treatment for vulvodynia; in fact, the available options (such as oral drugs, topical anesthetics, nerve infiltrations or botulinum toxin or even complementary medicine therapies such as acupuncture) are used only for symptomatic purposes to improve quality of life, without modifying the course of the disease [10]. Given its impact on sexual and mental health, a multidisciplinary approach that includes psychological or behavioral therapy and physiotherapy of the pelvic floor, both with favorable results in pain control and functionality, is recommended [9].

Clinical trials and systematic reviews that have been conducted in recent years highlight the problems of underdiagnosis, misdiagnosis, and psychological treatment [7,8]. Performing a meta-analysis on the subject becomes difficult because of the heterogeneous nature of the reviews and clinical trials carried out [11]. The lack of evidence concerns not only the therapeutic but also the tools for measuring the results of studies on vulvodynia [12]. In addition, the lack of criteria and standardized measurement tools hinders the objective evaluation of treatments and contributes to the persistent knowledge gap [11,12]. Although other reviews have analyzed vulvodynia as a starting point, quality of life has not been one of the objectives of these studies [3,5].

Thus, the general objective is to analyze the available scientific evidence on the quality of life of women with vulvodynia. In addition, the specific objectives are to evaluate the results of studies that analyze the quality of life of women diagnosed with vulvodynia, considering the different physical, psychological and social dimensions. On the other hand, it seeks to compare the tools and measurement scales used to evaluate the quality of life in this population and analyze their frequency of use, validity, and clinical applicability. Finally, it aims to explore the relationships between quality of life and sociodemographic or clinical factors, such as age, lifestyle, the presence of comorbidities and mental health.

## 2. Materials and Methods

### 2.1. Study Design

A quick review was conducted aimed at synthesizing the available evidence on quality of life among women diagnosed with vulvodynia.

To ensure methodological consistency with this type of design, the process was developed following the guidelines of the PRISMA 2020 Statement [13] (Supplementary Material Table S4) and the Interim Guide of the Cochrane Rapid Reviews Methods Group [14].

Consequently, the iterative decision points were previously defined, the differences with respect to a traditional systematic review were detailed, and the participation of knowledge users was documented to guarantee the applicability of the results.

All Supplementary Materials, including search and data extraction strategies, are provided as supplementary open-access documents to ensure reproducibility (Supplementary Material Table S1).

This review aims to answer the following research question: In women diagnosed with vulvodynia, how is their quality of life related to the diagnosis?

### 2.2. Eligibility Criteria

Inclusion criteria

- Primary studies (quantitative, qualitative or mixed methods).
- Unlimited publication date.
- Female population diagnosed with vulvodynia or vestibulodynia.
- Studies that include results related to quality of life, either through generic or specific instruments.

Exclusion criteria

- Reviews, editorials, comments, letters or conference abstracts without full text or primary data.
- Studies focused exclusively on treatment efficacy.
- Animal research.
- Case series with fewer than five participants when they do not provide validated measures.

### 2.3. Sources of Information and Search Strategy

The literature search was conducted in the PubMed, Web of Science (WOS), Scopus, Biblioteca Virtual en Salud (BVS), CINAHL and CUIDEN databases, without geographical or temporal restrictions.

In PubMed, the MeSH terms “Vulvodynia” and “Quality of Life” were used as the main descriptors, combined with the Boolean operator AND. In the rest of the databases, these same terms were used together with synonyms and textual variants to expand the retrieval of results: “Vestibulodynia”, “Generalized Vulvodynia”, “Vulva Pain”, “Pain, Vulva”, “vulvar pain”, as well as the equivalents of quality of life (“Life Quality”, “Health-Related Quality of Life”, “Health Related Quality Of Life”, “HRQOL”).

### 2.4. Data Extraction and Synthesis

The data were extracted using a standardized template that included the study design, country, sample size, objective of the study, instruments used to measure quality of life and main results.

Owing to the rapid nature of the review and the heterogeneity of the included studies, the results were synthesized in narrative form and organized into thematic domains and measurement tools.

### 2.5. Assessment of Quality and Risk of Bias

The methodological quality and risk of bias of the included studies were evaluated using the Mixed Methods Appraisal Tool. This validated instrument allows the systematic evaluation of both qualitative and quantitative studies (randomized, nonrandomized and descriptive) and mixed methods studies [15]. For each study, the five specific criteria corresponding to its type of design were applied, complemented by two initial screening questions. The studies were subsequently classified according to the number of criteria met, providing a structured and comparable estimate of methodological quality. The detailed results at the item level are presented in the Supplementary Material, along with the complete MMAT evaluation tables.

## 3. Results

**Figure 1:**
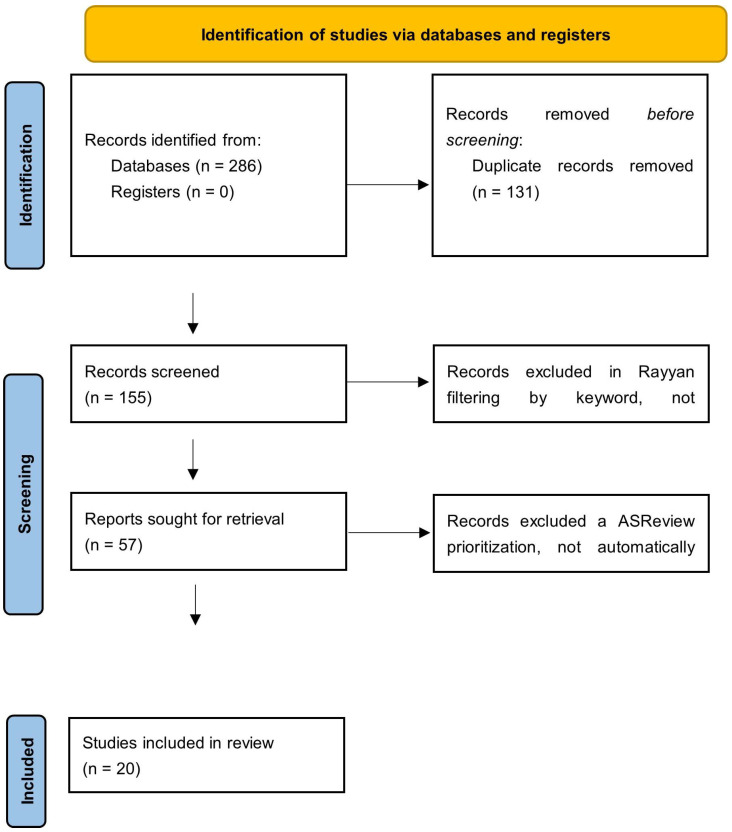
PRISMA 2020 flowchart showing the process of identification, screening and selection of the studies included in the review.

The process of the identification and selection of studies is illustrated in Figure 1, which shows the PRISMA 2020 flowchart that summarizes the process of the identification, screening, exclusion and final selection of the studies included in this review. In total, 286 records were retrieved from the selected databases: PubMed (*n* = 63), Web of Science (*n* = 64), Scopus (*n* = 100), BVS (*n* = 10) and CINAHL (*n* = 49). No additional records were identified in CUIDEN or in other complementary sources.

After the results were exported and integrated in the software Rayyan^®^ (Qatar Computing Research Institute) [16], duplicates were eliminated, and 131 repeated references were identified and deleted. The refined set of 155 unique records was subjected to screening by title and abstract, which was performed by three independent reviewers at Rayyan^®^. In this phase, 98 records were excluded, mainly because they did not address the quality of life associated with vulvodynia as the main topic.

The remaining 57 articles were subsequently prioritized by the ASReview^®^ tool (Maastricht University) [17] to improve the organization of the screening. This application allowed the references to be sorted according to their relevance, but the exclusion decisions remained manual and agreed upon by the reviewers. After this prioritization, 37 records were excluded because they did not meet the full-text eligibility criteria. Finally, 20 studies were included in the final review.

Both Rayyan^®^ and ASReview^®^ were used exclusively as support tools for the management, organization and prioritization of the search, without automating the decisions of inclusion or exclusion. All discrepancies were resolved by consensus among the three reviewers, ensuring the transparency and reproducibility of the process.

### 3.1. General Characteristics of the Included Studies

The twenty studies included in this review cover the period from 2006 to 2025, since there were no time restrictions. Thirteen quantitative studies and 7 qualitative studies were filtered out. In most quantitative studies, quality of life is evaluated using validated instruments.

Table 1 summarizes the main results of the quantitative articles analyzed, and Table 2 summarizes the qualitative results.

### 3.2. Assessment of Methodological Quality and Risk of Bias

The evaluation of methodological quality and risk of bias, performed using the Mixed Methods Appraisal Tool (MMAT, version 2018) [15], revealed that the overall quality of the included studies was satisfactory. Table 1 and Table 2 present, along with the general information of each of the documents included in the review, a summary of the overall analysis of methodological quality, while the detailed evaluation of each criterion is included in the Supplementary Material.

Quantitative studies, which represented most of the analyzed sample, showed moderate to high compliance with the methodological validity criteria. In general, the research objectives were clearly defined, the measurement instruments were appropriate for the phenomenon of study, and in most cases, validated scales were used for the evaluation of quality of life. However, recurring methodological risks associated with nonprobabilistic sampling, common in low-incidence pathologies such as vulvodynia, as well as the dependence on self-reported measures and the absence of control of confounding factors, were identified.

The qualitative studies, however, showed high methodological quality, with adequate coherence between the data, the methods of analysis and the interpretation of the results.

### 3.3. Assessment of Vulvodynia from the Physical, Psychological and Social Dimensions

#### 3.3.1. Physical Dimension

In the studies analyzed, the central element is persistent vulvar pain, which affects daily life [18,23,28,29]. Women report chronic pain that limits activities such as sitting or exercising, affecting their physical autonomy [23,29]; this pain directly interferes with daily tasks and functionality, generating a feeling of constant fatigue [23] and a global deterioration of perceived well-being, even in the absence of other gynecological pathologies [18]. Intense pain limits autonomy and is perceived as a force that dominates the body [36] and is aggravated by fear and a lack of control [22].

This pain conditions daily habits such as dressing, exercising or having sexual relations, generating behavioral avoidance [20,35], dyspareunia and physical discomfort associated with intercourse constitute important limitations [31]. Pain is related to lower resilience and worse sexual functioning [32]. Although the pain persists, its physical impact decreases when there is empathy and understanding in the couple [26].

Most patients experience localized pain and muscle dysfunction of the pelvic floor, with notable repercussions on their mobility and physical role [19,25]. The approach to vulvodynia is usually multimodal, combining physiotherapy, topical treatments, oral pharmacotherapy and psychological strategies; however, the results are partial, with improvements in functional disability and quality of life but the persistence of vulvar pain after six months of follow-up [27]. Although physiotherapy has been shown to improve bodily pain and physical function, it has not achieved complete resolution of symptoms [21]. In the same sense, patients report better control of pain and anxiety through mindfulness-based interventions, although they do not achieve total remission of symptoms [33].

In addition to the fact that the physical burden of pain was translated into high health costs, the disease entailed significant indirect costs, such as loss of employment or productivity [24].

#### 3.3.2. Psychological Dimension

Vulvodynia is associated with significant psychological effects, characterized by high levels of anxiety, depression, stress and emotional distress, as well as difficulties in coping with pain [18,23,24,29]. Compared with women with other chronic pathologies, women with vulvodynia have greater psychological distress and worse emotional quality of life [18,23,24]. In this sense, the presence of frequent psychiatric comorbidities, mainly anxiety, depression and chronic stress, contributes to the worsening of symptoms and the perception of greater disability [19,20,28,31]. An association between persistent pain and poor mental health was identified [19].

Stress acts as a pain amplifying factor, establishing a bidirectional relationship between emotional experience and the intensity of physical symptoms [29,30]. Similarly, cognitive factors such as catastrophizing, hypervigilance, and fear of pain are associated with an increase in pain intensity and a deterioration of psychological well-being [22].

Women report guilt, shame, frustration, low self-esteem, fear of pain, and feelings that negatively affect their well-being and self-image [31,34,36]. A lack of medical solutions generates feelings of frustration, hopelessness [35] and life stress [20].

The multimodal therapeutic approach, which includes physiotherapy, psychotherapy and body education strategies, has been shown to improve pain-related anxiety and emotional limitations, although it does not achieve complete resolution of symptoms [21,27]. Likewise, interventions with a positive impact on psychological adjustment, such as mindfulness, have been identified [33]. In addition, relational variables also affect psychological well-being: greater empathy and communication in the couple are associated with better emotional adjustment and quality of life [26].

Women with vulvodynia show greater anxious and avoidant attachment, lower resilience and more depressive symptoms than women without pain do [30,32].

On the one hand, the disease is associated with a crisis of identity and loss of femininity [34] and catastrophism and fear amplify pain and sexual dysfunction [22]. And on the other hand, perceived empathy improves emotional satisfaction and quality of life [26], and mindfulness and self-compassion promote acceptance and reduce psychological distress [33].

#### 3.3.3. Social and Relational Dimension

In the social and relational sphere, the results show a notable decrease in social participation and the quality of relationships [18,19,21,23,28,29], with a direct effect on social interaction [29,30] and difficulties in maintaining normal social or work routines [18,23]. Physical limitations directly affect social interaction, leading to isolation [19,28]. Similarly, the coexistence of comorbidities also restricts social life [19]. Physical improvement facilitates partial reintegration into social life [21]. Family and peer support are protective factors against isolation [33,36]. In the couple, empathy and open communication are determinants of preserving relational and emotional quality [26].

Women with vulvodynia have lower sexual satisfaction and worse adjustment to a partner, accompanied by feelings of frustration [31,32]. Vulvodynia affects intimacy and the avoidance of sexual contact [22]. Cultural norms about sexuality intensify relational suffering [34].

Vulvodynia alters social role and interpersonal relationships, especially in work or family contexts [25,27]. A lack of medical and social understanding aggravates stigma and withdrawal [20].

#### 3.3.4. Comparison of Measurement Tools and Scales Used

The studies included in this review show great heterogeneity in the tools used to assess the quality of life of women with vulvodynia. Thus, quantitative studies identify specific scales for measuring quality of life, and other specific scales focus on certain domains, such as sexual function or dermatological impact.

Several studies included in this review used validated instruments for the evaluation of quality of life, both generic and specific, to capture the multidimensional impact of vulvodynia. Among the generic questionnaires, the SF-12 [25,27,30] and the SF-36 [21] stand out because they provide summarized measures of physical and mental health. Similarly, the WHOQOL-BREF (World Health Organization) [29] and the EQ-5D (EuroQol) [24] provide global assessments of the perception of health and well-being, covering physical, psychological, social and environmental domains. With respect to the specific instruments used, the Sexual Quality of Life Questionnaire–Female (SQoL-F) [31] evaluates the quality of female sexual life, whereas the Ladder of Life Scale [18] reflects the subjective perception of general well-being. In addition, in dermatological profile studies, the Dermatology Life Quality Index (DLQI) [20,28] and the Skindex-29 [23,26] were applied, adapted for women with vulvodynia, both aimed at measuring the impact of symptoms and their emotional and functional repercussions. Finally, some studies incorporated measures of global self-perception of quality of life (Self-Reported QoL) [19,25], complementing the objective assessment with the subjective experience of the participants. Extensive heterogeneity is observed in the assessment tools used to assess the quality of life of patients with vulvodynia. Table 3 summarizes the scales used in the articles and provides a brief description of them.

Some studies have performed multidimensional measurements, evaluating aspects such as pain, sexuality, and aspects related to mental health. Table 4 summarizes the scales used based on the field explored.

#### 3.3.5. Exploration of Associated Sociodemographic and Clinical Factors

Taken together, the results of the included studies reveal remarkable homogeneity in the sociodemographic profiles of women affected by vulvodynia, although with some geographical and cultural variations. Most studies describe young or middle-aged women aged between 20 and 50 years, with the group of 30 to 40 years predominating as the most affected [18,19,21,23,24,25,27,28,29,31,32,33,37]. Larger population studies confirm that the maximum prevalence is concentrated in women under 40 years of age, although the disorder can persist or begin even after menopause [19,25,27].

Most of the participants were married or in a stable relationship [19,23,24,25,26,27,31,36], which allowed an analysis of the influence of the emotional support and empathy of the partner on quality of life.

In terms of educational level, most women with a diagnosis of vulvodynia have a middle or high educational level [18,20,24,25,27,29,32,33,35,36]. These findings may reflect a greater ability to access specialized health services and not necessarily a higher incidence of the disorder in this group. However, some studies (especially qualitative) suggest that women with lower educational levels may face greater difficulties in obtaining a correct diagnosis and timely medical care [35,36].

In terms of employment and economic situations, a loss of labor productivity and an increase in personal health costs associated with vulvodynia have been identified [24,27]. Chronic pain and emotional discomfort generate absenteeism and reduce working hours and job abandonment [35,36,37].

The most frequent clinical history was chronic pelvic pain, recurrent vulvovaginal infections, and psychological comorbidities such as anxiety or depression [18,19,25,28,30,32]. Some studies have identified factors of psychological vulnerability, such as adverse childhood experiences and anxious personality traits, that are associated with a worse perception of well-being [22,30].

Several studies included in this review revealed a high prevalence of medical and psychological comorbidities in women with vulvodynia, which reinforces the multifactorial nature of the disorder. In the physical realm, frequent associations are observed with chronic pain syndromes such as fibromyalgia, chronic fatigue syndrome, and irritable bowel or interstitial cystitis, which share mechanisms of central sensitization and autonomic nervous system dysfunction [19,30]. In the psychological sphere, depression, anxiety and chronic stress stand out as the most common comorbidities, which not only coexist with pain but also aggravates its perception and reduces quality of life [20,22].

Table 5 summarizes the sociodemographic factors presented in the articles.

## 4. Discussion

The studies reviewed indicate that compared with other vulvar pathologies, vulvodynia is a chronic condition characterized by persistent vulvar pain that significantly affects the quality of life of women [18,19,23,24,28,29].

Vulvodynia should be approached from a multidisciplinary perspective, recognizing the interaction between pain, mental health and interpersonal relationships [21,27]. The presence of persistent pain, burning and irritation can become so disabling that it prevents even sitting still for long periods, affecting daily activities and sexual relations. Several studies have shown that these conditions result in notable deterioration in different aspects of well-being [18,19,21,22,23,27,28,29].

The most recent evidence reinforces these conclusions. In 2025, Lountzi et al. emphasized that vulvodynia simultaneously compromises the physical, psychological and social spheres, generating a pattern of suffering comparable to that of other chronic pain syndromes [3]. Similarly, Rosen et al. reported that vulvodynia affects psychological health, sexual function, and interpersonal relationships; however, a comprehensive approach is needed [38]. More than 60% of Andrews report severe functional limitations and loss of control over their body, which affects their self-esteem and sexual life [39].

### 4.1. Dimensions Affected

Vulvodynia is a complex health problem with significant repercussions for the quality of life of women who suffer from it. Several studies have shown that this chronic vulvar pain disorder affects both physical well-being because of the persistence of pain and functional limitations [23,29] and psychological well-being because of the presence of anxiety, depression, stress and catastrophizing [20,22,30]. At the social and relational level, the effects on intimacy and sexual satisfaction, as well as on couple dynamics, are evident. Empathy and communication act as protective factors [26,32].

Recent literature agrees with this approach. Persistent pain and vulvar hypersensitivity limit basic activities and sexual life, aggravating the perception of social isolation [3]. The presence of anxiety, depression, and catastrophizing not only accompanies pain but also amplifies its intensity and reduces sexual satisfaction [5]. In addition, local inflammatory processes and peripheral sensitization contribute to the maintenance of pain and psychological deterioration [40].

In the physical realm, the reduction in the ability to perform daily activities is highlighted [21,23,24,27,28,29]. On the other hand, we find fewer articles that assess sexual function [25,27], although in general, a marked decrease in sexual satisfaction and desire is mentioned, as well as a high prevalence of dyspareunia [18,19,25,26,27,33,34]. The relationship between pain intensity and quality of life is directly proportional, which means that the greater the pain is, the worse the quality of life reported by the patient [27,28,29,30].

The emotional consequences of vulvodynia include feelings of frustration, anxiety, and severe depression, which accompany pain and sexual dysfunction and further aggravate the perception of quality of life [20,27,33,34,35,36,37]. Stress is directly correlated with increased pain perception, generating a vicious cycle that compromises the overall well-being of patients. This situation perpetuates itself: women anticipate pain during sexual contact, leading to reactive hypertonicity, increased sensitivity, and thus continuation of the pain cycle [20,22,27,29]. Chisari et al. [5] and Rosen et al. [38] support this feedback model between psychological factors (anxiety, fear of pain and catastrophizing) and the persistence of pain, highlighting the need for interventions that modify cognitions and emotions related to pain [5,38].

Several studies included in this review revealed a high prevalence of medical and psychological comorbidities in women with vulvodynia, which reinforces the multifactorial nature of the disorder. In the physical realm, frequent associations are observed with chronic pain syndromes such as fibromyalgia, chronic fatigue syndrome, and irritable bowel or interstitial cystitis, which share mechanisms of central sensitization and autonomic nervous system dysfunction [18,30]. In the psychological sphere, depression, anxiety and chronic stress stand out as the most common comorbidities, which not only coexist with pain but also aggravates its perception and reduces quality of life [20,22]. The presence of comorbidities such as recurrent infections, sensitivities or allergies in the vulvar area, in addition to a history of vulvar surgery or other pathologies such as candidiasis and urinary tract pathologies, also contributes to a worsening of quality of life and sexual function [18,19,24,25,26,27,33,34].

On the other hand, multidisciplinary interventions, including physical therapy, psychological therapy and pain management strategies, have proven useful for improving aspects of quality of life related to physical and emotional well-being, although a negative impact on sexual function still persists [21,23,25,27,35,37]. In this sense, Calafiore et al. [41] reported that rehabilitative therapies significantly reduce pain and improve physical quality of life, although the effects on the sexual and psychological spheres remain limited. In addition, the application of psychological therapies aimed at reducing stress and anxiety seems to be beneficial, since comorbidities such as depression are strongly associated with vulvodynia and affect the prognosis of quality of life [20,21,25,27,33,35,37].

Experiences of pain are better measured with qualitative studies since they allow the experience of these women to be understood [33,34,35,36,37]. Lountzi et al. [3] and Andrews [39] agreed that qualitative approaches provide a more comprehensive view of emotional and social impact, complementing the quantitative information on pain and function [3,39].

### 4.2. Quality of Life Measurement Scales

The available evidence shows marked heterogeneity in the results on quality of life, both in the dimensions evaluated and in the magnitude of the reported impact, attributable to methodological and cultural differences and in the instruments used, which makes direct comparison between studies difficult [18,19,21,23,26,27,28,29,32,33,35,37]. Lountzi et al. [3] confirm this lack of uniformity, identifying the use of generic instruments (SF-12, SF-36, and WHOQOL-BREF) along with specific scales such as the FSFI and the VAS, and underscore the need to standardize measures to improve the comparability of the results [3]. Chisari et al. [5] reported that the inclusion of psychological scales such as the HADS, PCS or BDI is essential for capturing the influence of emotions on pain and quality of life [5]. Similarly, the combined use of multidimensional tools that integrate physical, psychological, and relational domains is recommended [38].

### 4.3. Sociodemographic Factors

Vulvodynia affects mainly young or middle-aged women (30–40 years) with medium-high educational levels and is associated with multiple chronic comorbidities, such as migraine, irritable bowel syndrome, fibromyalgia, anxiety and depression [20,24,25,29,30,35].

Other studies support this epidemiological profile, which is consistent with the presence of a medium–high educational level and a history of chronic pain or persistent stress [3], in addition to being with women of reproductive age [39]. The coexistence of inflammatory and immunological processes with hormonal and psychological factors explains the clinical variability and the unequal response to treatment [40].

## 5. Limitations

Several limitations of the present review should be considered; the main limitation is the heterogeneity of the study designs, with a predominance of descriptive studies, which limits the possibility of establishing causal relationships between the clinical and psychological variables and the results of a study life. The representativeness of the samples is also a limitation, given that most of the research was conducted in clinical settings or with recruitment by convenience, mainly reflecting the experience of women seeking medical care. In addition, many studies have been based on self-reports of symptoms and measures of well-being, which could introduce biases in recall or social desirability, especially in sensitive domains such as sexual function or the perception of one’s own body.

Although validated instruments have been used to measure quality of life, the diversity of tools used (SF-12, WHOQOL-BREF, DLQI, Skindex-29, FSFI, among others) makes comparisons between studies difficult. quantitative synthesis of results. However, being able to analyze quality of life from both qualitative and quantitative studies allows a broad, although very general, view.

The absence of controlled clinical trials that specifically evaluate quality of life is explained, in part, by the fact that experimental research in vulvodynia tends to focus on the efficacy of specific treatments and does not include these results as the main objective, which has limited their inclusion in this review.

## 6. Conclusions

The evidence analyzed confirms that vulvodynia is a complex chronic condition that significantly affects the quality of life of women, with interrelated physical, psychological and social repercussions. Persistent pain, anxiety, depression and functional and relational limitations form a pattern of biopsychosocial suffering comparable to that of other chronic pain syndromes.

Important methodological heterogeneity is detected regarding the evaluation of the quality of life of women. Generic scales of quality of life associated with pain assessment are used, although there is a marked trend toward a comprehensive evaluation, incorporating the use of psychological and sexual function questionnaires. This high variability in the analysis makes comparisons between investigations difficult and reinforces the need for a consensus on standardized measures of analysis in patients with vulvodynia, adapted to their biopsychosocial nature.

A pattern is observed within the sociodemographic and clinical factors, where they are affected mainly by young and middle-aged women, with medium-high educational levels, and with a frequent association of comorbidities such as fibromyalgia, irritable bowel syndrome, interstitial cystitis, migraine and mood disorders. This finding supports the need for a comprehensive and multidisciplinary approach that recognizes the interactions among the biological, psychological, and social determinants of pain.

## Figures and Tables

**Table 1:** Included Quantitative Studies (*n* = 13).

Author and Year	Country	Design/Sample	Objective of the Study	QoL Instruments	Main Findings on Quality of Life	Level of Evidence/Quality
Arnold et al., 2006 [18]	U.S.	Case–control survey (*n* = 382)	To compare health history and health care utilization habits of women diagnosed with vulvodynia to that of an asymptomatic control group	Ladder of Life Scale	It is shown that vulvodynia impacts quality of life and sexual health, in addition to presenting comorbidities such as fibromyalgia or irritable bowel syndrome.	High-moderate
Arnold et al., 2007 [19]	U.S.	Case–control study; a phone survey (*n* = 100 symptomatic women; *n* = 325 asymptomatic women)	To evaluate the prevalence of symptoms of vulvodynia in a representative sample of American women and to compare the health characteristics of symptomatic and asymptomatic women	Self-reported quality of life	Negative impact on physical and mental health; frequent comorbidities and reduced quality of life.	High
Tribó et al., 2008 [20]	España	Observational, longitudinal and descriptive study (*n* = 80)	To evaluate the main clinical signs associated with psychopathological disorders and outcome after antidepressant treatment of patients with vulvodynia	Dermatology Life Quality Index (DLQI)	There is a high prevalence of anxiety and depression with worse quality of life; Clinical improvement is detected after combined treatment with antidepressants	High
Forth et al., 2009 [21]	United Kingdom	Quasiexperimental (*n* = 21; 14 started and completed the study)	To evaluate the use of physiotherapy in patients with vulvodynia.	SF-36	Patients manifested pain relief with physiotherapy, but no statistically significant differences were detected due to the small sample size.	High-moderate
Desrochers et al., 2009 [22]	Canada	Transversal (*n* = 75)	To analyze psychological variables associated with pain and sexual dysfunction.	Indirect measurement from pain, sexual function and psychological variables	Greater catastrophization, fear of pain, hypervigilance and low self-efficacy are associated with greater pain intensity and sexual dysfunction.	High
Ponte et al., 2009 [23]	U.S.	Cross-sectional comparison (*n* = 101 patients with vulvodynia; 179 patients with other vulvar conditions)	To determine the impact of vulvodynia on quality of life compared to other skin disorders and other vulvar conditions.	Skindex-29	Patients with vulvodynia showed a significantly lower quality of life and greater psychological and physical comorbidity compared to other vulvar disorders	High-moderate
Xie et al., 2012 [24]	U.S.	Longitudinal descriptive (*n* = 302; *n* = 97 completed the data for 6 months)	To estimate the economic burden and its relationship with quality of life in women with vulvodynia	EQ-5D	There is a strong deterioration of the quality of life and implies a significant economic burden.	Moderate
Lamvu et al., 2015 [25]	U.S.	Prospective cohort registry (*n* = 323 women were enrolled and *n* = 250 were followed)	To create a National registry for the study of vulvodynia in order to enhance classification of vulvodynia based on multiple phenotypic domains	SF-12	Overall impact on quality of life; high prevalence of comorbidities, muscle dysfunction and emotional distress.	High-moderate
Rosen et al., 2016 [26]	Canada	Observational (*n* = 50 pairs)	To investigate the observed and perceived associations between disclosure and empathic response, and couples’ relationship adjustment, as well as women’s pain during intercourse, and quality of life	Skindex-29	Greater empathy and open communication in the couple was associated with better relational adjustment and higher quality of life, without significant changes in pain intensity	High
Lamvu et al., 2018 [27]	U.S.	Prospective cohort (*n* = 282)	To describe treatment patterns and clinical outcomes at six months in women with vulvodynia enrolled in the National Vulvodynia Registry	SF-12	Improvements were detected after six months in pain, physical function, emotional well-being and quality of life, although sexual function continued to deteriorate.	Moderate
Tribó et al., 2020 [28]	Spain	Cross-sectional observational study (*n* = 110)	to determine the characteristics of pain in vulvodynia, to correlate characteristics with symptoms of anxiety and depression, and to analyze the impact of these factors on patients’ quality of life.	Spanish version of the Dermatology Life Quality Index (DLQI)	Most patients had severe pain related to psychiatric comorbidities and decreased quality of life.	High-moderate
Patla et al., 2023 [29]	Poland	Cross-sectional (*n* = 76 women)	To evaluate the severity of pain and its impact on health-related quality of life.	WHOQOL-BREF	Pain was more intense in young women and with medium or high levels of stress, negatively affecting quality of life, especially daily activities and sexual life	Moderate
Nimbi et al., 2024 [30]	Italy	A cohort-based cross-sectional web survey (*n* = 357)	To explore the role of psychological factors (temperament, personality traits, adverse childhood experiences, defense mechanisms and mental pain) in central sensitization and quality of life of women with vulvodynia	SF-12	Central sensitization and mental pain explain more than 50% of the variance in quality of life.	High-moderate
Çankaya & Meler, 2025 [31]	Turkey	descriptive, correlational, and comparative study (*n* = 220 women with and without vulvodynia)	To determine genitourinary pain, sexual distress, and quality of sexual life of women with and without vulvodynia.	Sexual Quality of Life Questionnaire-Female (SQLQ-F)	Women with vulvodynia had greater genitourinary pain, greater sexual distress and worse sexual quality of life compared to women without vulvodynia.	High
Gattamelata et al., 2025 [32]	Italy	Cross-sectional comparison (A total of 203 women; *n* = 96 with vulvodynia: *n* = 107 controls)	To explore the psychological, relational, and fertility-related characteristics of women with vulvodynia	Indirect measurement through multidimensional scales	Worse sexual function, lower resilience and adjustment of the couple; greater distress related to fertility.	High

**Table 2:** Qualitative Studies Included (*n* = 7).

Author and year	Country	Design/Sample	Objective of the Study	Approach/Method	Main Findings on Quality of Life	Level of Evidence/Quality (MMAT)
Brotto et al., 2013 [33]	Canada	Phenomenological (*n* = 14)	To explore the effects of treatment based on mindfulness and cognitive behavioral therapy.	Thematic content analysis	Mindfulness improves acceptance and well-being; sustained increase in perceived QL.	High
Groven et al., 2015 [34]	Norway	Phenomenological (*n* = 8)	To explore the lived experience of women with vestibulodynia.	Hermeneutic phenomenological analysis	Pain as a threat to femininity; loss of identity; Body acceptance improves quality of life.	High
LePage & Selk, 2016 [35]	Canada	Grounded theory (*n* = 8)	Identify unmet needs and perceptions about clinical care.	Constant comparative method	Pain and therapeutic barriers reduce physical and emotional QoL; demand for a multidisciplinary approach.	High
Montali et al., 2025 [36]	Italy	Cualitativo (*n* = 35)	To explore protective and aggravating factors of the experience with vulvodynia.	Reflective thematic analysis	Pain and lack of medical examination deteriorate QoL; family support and resilience improve it.	High
Harryson & Sjöström, 2025 [37]	Sweden	Qualitative content analysis (*n* = 10)	To investigate the felt and known experience of living with provoked vulvodynia in a group of women in Sweden.	Inductive content analysis	Women emphasized that understanding their body and the purpose of treatment is essential. The participation of the professional in the initial phase of treatment facilitates a faster diagnosis and a better therapeutic response.	High

**Table 3:** Quality of life measurement tools used in the included studies.

Instrument/Scale	Articles that Use It	Main Results Obtained
SF-12 (Short Form-12 Health Survey)	[25,27,30]	Derived from the original SF-36, is a concise generic health survey designed to gauge physical and psycho-logical QoL. Enhanced scores reflect a higher level of QoL in the indicated domain.
SF-36	[21]	Covers eight aspects of health status: physical functioning, physical role, bodily pain, general health, vitality, social functioning, emotional role and mental health. Scores in each aspect are transformed into scales which range from 0 to 100.
WHOQOL-BREF (WHO) The World Health Organization Quality of Life (WHOQOL-BREF) Questionnaire	[29]	Consists of 26 questions that assess respondents’ perception of quality of life, perception of their own health, and quality of life in four domains: physical health, psychological, social, and environmental.
EQ-5D (EuroQol)	[24]	Five attributes (Mobility, Self-Care, Usual Activities, Pain/Discomfort, and Anxiety/Depression) were rated on three levels.
SQLQ-F (Sexual Quality of Life-Female)	[31]	Each item in the scale is expected to be answered by considering the quality of women’s sexual life in the previous four weeks. The scale has 18 six-point Likert-type items.
Ladder of Life Scale	[18]	Reduction in perceived general well-being; correlation with intensity of chronic pain.
DLQI (Dermatology Life Quality Index)	[20,28]	10 items with a temporary frame of the last 7 days. The health dimensions included are symptoms and perceptions, daily activities, leisure time, work/studies, interpersonal relationships and sexuality, and treatment.
Skindex-29	[23,26]	A measure of quality of life for those with skin diseases. It was adapted for use in women with vulvodynia: 15 items adapted from the Skindex-29 as well as three additional items were included to assess the emotional and functional dimensions of women’s quality of life during the previous four weeks.
Self-reported quality of life	[19]	Overall subjective assessment The reduced quality of life in this population underscores the need for comprehensive assessment and multidisciplinary care.
Qualitative analysis	[33,34,35,36,37]	Qualitative analysis, subjective evidence. It refers to the expressions of women during the qualitative interview.

**Table 4:** Other scales used in the included studies.

Instrument (Acronym)	Articles	Description
**Pain/Intensity**		
VAS (Visual Analog Scale)	[20,25,28,29]	Line of 10 cm between ‘no pain’ and ‘worst imaginable pain’ to mark pain intensity.
PIVS (Pain Intensity Verbal Scale)	[20]	Five descriptors: mild, moderate, severe, very severe and unbearable.
MPQ (McGill Pain Questionnaire)	[20,21,22,25,27,28]	Classic questionnaire of 3 categories (sensory, affective, evaluative); reference in pain measurement.
PPI/NRS (Present Pain Intensity/Numeric Rating Scale)	[22,26]	Scale of 0–10 to measure the average pain during intercourse in the last six months.
VPI (Vestibular Pain Index)	[22]	Evaluates pain induced during gynecological examination in different vestibular areas (0–10).
GPS (Gracely Box Pain Scale)	[25,27]	It measures sensory and affective components of pain related to intercourse.
SPP (Static Pressure Pain Threshold)	[27]	Determines the pain pressure threshold by cotton test in vestibular mucosa.
VRS (Verbal Rating Scale)	[28]	Verbal scale with five degrees of pain intensity, from mild to unbearable.
F-GUPI (Female Genitourinary Pain Index)	[31]	Evaluates pelvic pain and genitourinary symptoms in the last six months.
**Sexuality/Sexual function**		
FSFI (Female Sexual Function Index)	[22,25,27,31,32]	19 items, six dimensions of sexual function (desire, arousal, lubrication, orgasm, satisfaction and pain).
FSDS (Female Sexual Distress Scale)	[31]	13 items that assess sexual distress during the last four weeks.
Painful Intercourse Self-Efficacy Scale	[22]	20 items that measure self-efficacy in managing pain during intercourse.
FPI-SF (Fertility Problem Inventory–Short Form)	[32]	It evaluates stress and the social, sexual and relational impact of fertility problems.
**Psychological/Emotional**		
BDI (Beck Depression Inventory)	[27,27,32]	21 items to detect and quantify depressive symptoms.
HADS (Hospital Anxiety and Depression Scale)	[20,28]	Screening scale for anxiety and depression in the nonpsychiatric population.
HRSA (Hamilton Rating Scale for Anxiety)	[20,28]	14 items scored from 0 (absent) to 6 (very severe); measures severity of anxiety.
STAI (State Trait Anxiety Inventory)	[22,25,27]	40 items; evaluates state-anxiety and trait-anxiety.
LES (Life Event Scale)	[20]	43 stressful life events representative of triggering situations.
Mental Pain Questionnaire	[30]	Evaluates emotional pain or psychological suffering as a subjective state of anxiety.
**Relational/Social**		
AS-7 (Dyadic Adjustment Scale)/ DAS-4 (abbreviated version)	[32] [25,26]	Measures the quality of adjustment and satisfaction in the couple (consensus, cohesion and satisfaction). DAS-4: Four items on frequency of disagreement and perceived happiness.
ECR-12 (Experience in Close Relationship Scale–12)	[32]	Self-reported 12-item questionnaire that evaluates patterns of adult attachment in couple relationships. It includes two dimensions: avoidance and attachment anxiety.
**Personality/Coping**		
RS-14 (Resilience Scale)	[32]	14 items on ability to cope with adversity.
PCS (Pain Catastrophizing Scale)	[22]	Divided into 3 subscales that assess different components of catastrophic thinking (rumination, magnification, helplessness).
PASS-20 (Pain Anxiety Symptoms Scale)	[22]	Abbreviated version (20 items) that measures fear and avoidance of pain.
CSQ (Coping Strategies Questionnaire)	[25,27]	Measures strategies for coping with pain, including catastrophism.
PVAQ (Pain Vigilance and Awareness Questionnaire)	[22]	It evaluates attention, vigilance and pain awareness.
ATQ (Approach-Avoidance Temperament Questionnaire)	[32]	Evaluates approach temperament and emotional avoidance.
CSI (Central Sensitization Inventory)	[30]	Detects overlapping symptoms of central sensitization syndrome.
**Central/Psychobiological Sensitization**		
Highly Sensitive Person Scale	[30]	Evaluates sensitivity to sensory stimuli and emotional burden.
Defense Mechanisms Rating Scales–Self-Report	[30]	Quantifies neurotic and immature defense mechanisms.
Personality Inventory for DSM-5 (Short Form)	[30]	Evaluates pathological personality traits according to the DSM-5.
Traumatic Experiences Checklist	[30]	29 types of traumas, including emotional, physical and sexual abuse.

**Table 5:** Sociodemographic and contextual factors associated with quality of life in women with vulvodynia.

Reference (Year)	Average Age/Range	Marital Status/Partner	Educational Level/Employment	Most Frequent Comorbidities/QoL Results	Symptoms
Arnold et al., 2006 [18]	43.1 ± 13.7	Married (*n* = 50; 64.9%) Single (*n* = 18; 23.4%)	-	Average Quality of life 7 ± 1.9 Stress self-reported in life 5.9 ± 2.3	Burning (*n* = 67; 88.2%) Itching (*n* = 46; 60.5%)
Arnold et al., 2007 [19]	35 (18–64)	Married (*n* = 69; 69%)	High School or less (*n* = 27; 27%); Some College (*n* = 26; 26%); College degree or Higher (*n* = 47; 47%) Employed (*n* = 69; 70.4%)	Mean QoL (8.4) Mean stress level (6.88) Depression (*n* = 46; 46%) Painful periods (*n* = 76; 76%)	Burning (67%); itching (55%); aching (43%). Perception of symptoms caused by stress (39%) and yeast infections (35%).
Tribó et al., 2008 [20]	46.9 ± 13.0	-	College degree (52.6%) At least 10 years of education (47.4%)	Generalized anxiety (*n* = 15; 18.7%) Depression-anxiety syndrome (*n* = 6; 7.5%) Somatizing disorder (*n* = 6; 7.5%)	Pain (*n* = 56; 70%); burning (*n* = 51; 63.7%); dyspareunia (*n* = 46; 57.5%); stinging (*n* = 45; 56.2%); itching sensations (*n* = 40; 50%).
Forth et al., 2009 [21]	31.71	-	-	SF-36 results: physical functioning (47.79 ± 11.82); physical role (41.50 ± 13.40); bodily pain (41.56 ± 8.06); general health (42.01 ± 11.44); physical summary (43.99 ± 11.34)	Main diagnosis: Vulvar vestibulitis (71.4%); Aesthetic vulvodynia (28.6%)
Desrochers et al., 2009 [22]	27 ± 6.1	Dating (*n* = 29± 38.7) Cohabiting with partner (*n* = 28 ± 37.3) Married (*n* = 10 ± 13.3) Single (*n* = 8± 10.7)	-	-	Pain duration (years) 5.7 ± 4.9
Ponte et al., 2009 [23]	42 ± 16	Married (*n* = 50; 51%) Single (*n* = 37; 37%)	-	Frequent urinary infections (*n* = 28; 29%); Frequent yeast infections (*n* = 64; 65%) Depression (*n* = 47; 47%)	Characteristics of vulvar pain: localized (*n* = 57; 61%); generalized (*n* = 36; 39%)
Xie et al., 2012 [24]	38.88 ± 13.31	Married (*n* = 177; 58,80%) Single (*n* = 57; 18.94%) Partner (*n* = 27; 8.97%) Engaged (*n* = 20; 6.64%) Divorced/separated (*n* = 17; 5.65%) Widowed (*n* = 3; 1%)	High school (*n* = 35; 11.59%) Associate degree (*n* = 36; 11.92%) Undergraduate degree (*n* = 114; 37.75%) Graduate degree (*n* = 96; 31.79) Graduate degree: MD, GM, etc. (*n* = 21; 6.95%)	Irritable bowel syndrome (*n* = 82; 27.15%) Migraine headache (*n* = 76; 25.17%) Temporomandibular disorders (*n* = 51; 16.89%) Interstitial cystitis (*n* = 41; 13.58%) Endometriosis (*n* = 35; 11.59%) Fibromyalgia (*n* = 32; 10.60%)	Subtype of vulvodynia: Generalized vulvodynia (*n* = 97; 32.12%); Vestibulodynia (*n* = 121; 40.07%); Both (*n* = 84; 27.81%) How long ago was the first diagnosis (months) 39.12–47.45 How long ago did the symptoms start (months) 85.59–90.78
Lamvu et al., 2015 [25]	32.7 ± 11.4	Married or in a stable relationship (*n* = 117; 67%) Single (*n* = 35; 19.8%)	Completed college (*n* = 74; 42.1%) Postgraduate Study (*n* = 53; 30.1%)	Migraine headaches (*n* = 50; 34%); chronic pelvic pain (*n* = 32; 22%); irritable bowel syndrome (*n* = 30; 20%)	VAS score >3 in 100%
Rosen et al., 2016 [26]	24.50 ± 4.03	100% in stable relationship: Cohabitating (*n* = 26; 52%) Committed (*n* = 21; 42%) Married (*n* = 3; 6%)	Education level (years) 15.92 ± 2.06 (12–22)		Women’s pain duration (months) 51.50 ± 43.34 (6–180)
Lamvu et al., 2018 [27]	34.1 ± 12.2	Married/in a relationship (*n* = 151; 53.55%)	Some college (*n* = 42; 14.9%) Completed college (*n* = 87; 30.9%) Graduate (*n* = 55; 19.5%) Employed (*n* = 136; 48.2%)	-	At least 1 treatment since diagnosis (*n* = 282) Median pain duration 24 months Treatments: topical (*n* = 241; 85%), physical therapy (*n* = 147; 52%), oral medications (*n* = 128; 45%). 73% of participants received ≥ 2 treatments
Tribó et al., 2020 [28]	43.4 ± 13.5	-	Employment status: active (*n* = 87; 79.1%)	Previous surgeries (*n* = 64; 58.7%) Known allergies (*n* = 45; 41.3%) Psychiatric comorbidities (*n* = 41; 37.3%)	Stinging (*n* = 75; 68.2%); Burning (*n* = 72; 65.5%); Pain (*n*= 69; 62.7%); Itching (*n* = 63; 57.3%)
Patla et al., 2023 [29]	30.93 ± 8.24	Married (*n* = 33; 43.42%) Single (*n* = 40; 52.63%)	Higher education (*n* = 53; 69.74%)	Bladder problems (*n* = 30; 39.47%); Jaw clenching, temporomandibular joint (*n* = 30; 39.47%); Intestinal problems (*n* = 26; 34.21%); Back pain (*n* = 26; 34.21%)	Never being pregnant (*n* = 50; 65.79%)
Nimbi et al., 2024 [30]	36.08 ± 12.67	Monogamous couple (*n* = 288; 80.67%) Single (*n* = 67; 18.76%) Non monogamous relationship (*n* = 2; 0.56%)	Education level: High school (*n* = 133; 37.25); University (*n* = 162; 45.37%); PhD and postgraduate courses (*n* = 48; 13.45%) Employed (*n* = 220; 61.62%)	Restless leg syndrome (*n* = 55; 15.40%); Chronic fatigue syndrome (*n* = 80; 22.40%); Chronic migraine and tensive headache (*n* = 95; 26.61%); Temporomandibular disorders (*n* = 87; 24.37%); Irritable bowel syndrome (*n* = 153; 42.86%); Fibromyalgia (*n* = 107; 30%)	-
Çankaya & Meler, 2025 [31]	35.2 ± 8.4	100% married/in a relationship	Primary school (*n* = 39; 58.2%); Bachelor (*n* = 47; 51.1%); High school (*n* = 24; 39.3%) Employed (*n* = 53; 47.7%); Full time housewife (*n* = 57; 52.3%)	Avoidance of sexual intercourse (*n* = 88; 60.7%); Pain or feeling of burning during sexual intercourse (*n* = 84; 75%)	Pain or discomfort (6.6 ± 4.2) Urination (1.6 ± 2.7) QoL (3.5 ± 2.7)
Gattamelata et al., 2025 [32]	31.9 ± 7.31	Married (*n* = 21; 21.9%) Single (*n* = 57; 59.4%)	University degree or higher (*n* = 50; 52.1%) High school (*n* = 44; 45,8%) Employed (*n* = 65; 67.7%)	-	-
Brotto et al., 2013 [33]	39.6 ± 13.6	In a relationship (*n* = 9; 64.29%)	Postsecondary education 100%	-	Lifelong provoked vestibulodynia (*n* = 6); Acquired (*n* = 8) N° of years they had provoked vestibulodynia (2–26)
Groven et al., 2015 [34]	-	In a relationship (75%)	-	-	Described symptoms: Burning, prickly, searing pain
LePage & Selk, 2016 [35]	18–24 (*n* = 3) 25–34 (*n* = 1) 35–44 (*n* = 1) 45–54 (*n* = 2) 55–64 (*n* = 1)	Married (*n* = 3; 38%) Single (*n* = 3; 38%) Divorced/separated (*n* = 2; 25%)	College (*n* = 3; 38%); High school (*n* = 1; 12%); Professional school (*n* = 2; 25%); University (*n* = 2; 25%) Employment: Student (*n* = 2; 25%); Employed (*n* = 6; 75%)	Depression (*n* = 3; 38%) Isolation (*n* = 3; 38%) Anger (*n* = 2; 25%) Frustration (*n* = 2; 25%) Anxiety (*n* = 1; 13%) Stress (*n* = 1; 13%)	
Montali et al., 2025 [36]	18–20 (*n* = 10; 28.58%) 20–30 (*n* = 9; 25.70%) 30–40 (*n* = 10; 28.58%) 40–50 (*n* = 6; 17.14%)	In a relationship (*n* = 31; 88.57%) Single (*n* = 4; 11.43%)	Bachelor’s/ Master’s degree (*n* = 23; 65.71%); High School Diploma (*n* = 12; 34.29%) Employment: Full-time/part-time (*n* = 23; 65.7%) Student (*n* = 12; 34.29%)	-	-
Harryson & Sjöström, 2025 [37]	30.4 (27–39)	-	-	-	Duration of symptoms in years 7.3 years (3–20 years) The average length of time to diagnosis was 5.2 years (1–19 years).

## Data Availability

No new data were created or analyzed in this study. Data sharing is not applicable to this article.

## Supplementary Materials

The following supporting information can be downloaded at: https://www.mdpi.com/article/10.3390/jcm15010070/s1, Table S1: Databases consulted and search processing. Table S2: Checklist for selection criteria of the articles. Table S3: Risk of bias evaluation-Qualitative studies. Table S4: PRISMA 2020 Checklist.

## References

[1] Nakhleh-Francis Y., Awad-Igbaria Y., Sakas R., Bang S., Abu-Ata S., Palzur E., Lowenstein L., Bornstein J. (2024). Exploring Localized Provoked Vulvodynia: Insights from Animal Model Research. Int. J. Mol. Sci..

[2] Mocini E., Donini L.M., Isidori A.M., Minnetti M. (2024). Nutritional and metabolic aspects related to vulvodynia: What do we really know?. Nutrition.

[3] Lountzi A.Z., Abhyankar P., Durand H. (2025). A scoping review of vulvodynia research: Diagnosis, treatment, and care experiences. Womens Health.

[4] Baszak-Radomańska E., Wańczyk-Baszak J., Paszkowski T. (2025). Pelvic floor examination in vulvodynia: VAMP protocol validation in correlation with central sensitization. Womens Health.

[5] Chisari C., Monajemi M.B., Scott W., Moss-Morris R., McCracken L.M. (2021). Psychosocial factors associated with pain and sexual function in women with Vulvodynia: A systematic review. Eur. J. Pain.

[6] Jackman V., Bajzak K., Rains A., Swab M., Miller M., Logan G.S., Gustafson D.L. (2024). Physical Modalities for the Treatment of Localized Provoked Vulvodynia: A Scoping Review of the Literature from 2010 to 2023. Int. J. Womens Health.

[7] Moravek M.B., Legocki L.J., Piper C.K., Bernard K., Reed B.D., Haefner H.K. (2023). Impact of a single-session psychosocial counseling intervention for women with vulvodynia. Int. J. Gynecol. Obstet..

[8] Bitzi G., Kokka I., Mourikis I. (2025). A systematic review on the efficacy of CBT on pain and sexual function of vulvodynia. Psychiatriki.

[9] Engström A.H., Bohm-Starke N., Kullinger M., Hesselman S., Högberg U., Buhrman M., Skalkidou A. (2022). Internet-based Treatment for Vulvodynia (EMBLA)—A Randomized Controlled Study. J. Sex. Med..

[10] Bajzak K., Rains A., Bishop L., Swab M., Miller M.E., Logan G.S., Jackman V., Jackman L., Gustafson D.L. (2023). Pharmacological Treatments for Localized Provoked Vulvodynia: A Scoping Review. Int. J. Sex. Health.

[11] Bohm-Starke N., Ramsay K.W., Lytsy P., Nordgren B., Sjöberg I., Moberg K., Flink I. (2022). Treatment of Provoked Vulvodynia: A Systematic Review. J. Sex. Med..

[12] Pukall C., Hellberg C., Österberg M., Jonsson A.K., Kempe S., Gustavsson P., Bohm-Starke N. (2025). Psychometric properties for instruments used to measure core outcomes for provoked vestibulodynia: A systematic review. J. Sex. Med..

[13] Page M.J., McKenzie J.E., Bossuyt P.M., Boutron I., Hoffmann T.C., Mulrow C.D., Shamseer L., Tetzlaff J.M., Akl E.A., Brennan S.E. (2021). The PRISMA 2020 statement: An updated guideline for reporting systematic reviews. BMJ.

[14] Stevens A., Hersi M., Garritty C., Hartling L., Shea B.J., Stewart L.A., Welch V.A., Tricco A.C. (2025). Rapid review method series: Interim guidance for the reporting of rapid reviews. BMJ Evid. Based Med..

[15] Hong Q.N., Pluye P., Fàbregues S., Bartlett G., Boardman F., Cargo M., Dagenais P., Gagnon M.-P., Griffiths F., Nicolau B. (2019). Improving the content validity of the mixed methods appraisal tool: A modified e-Delphi study. J. Clin. Epidemiol..

[16] Ouzzani M., Hammady H., Fedorowicz Z., Elmagarmid A. (2016). Rayyan—A web and mobile app for systematic reviews. Syst. Rev..

[17] ASReview LAB Developers (2025). ASReview LAB: A Tool for AI-Assisted Systematic Reviews.

[18] Arnold L.D., Bachmann G.A., Rosen R., Rhoads G.G. (2006). Vulvodynia: Characteristics and Associations with Comorbidities and Quality of Life. Obstet. Gynecol..

[19] Arnold L.D., Bachmann G.A., Rosen R., Rhoads G.G. (2007). Assessment of Vulvodynia Symptoms in a Sample of U.S. Women: Prevalence Survey with a Nested Case–Control Study. Am. J. Obstet. Gynecol..

[20] Tribó M.J., Andión O., Ros S., Gilaberte M., Gallardo F., Toll A., Ferrán M., Bulbena A., Pujol R.M., Baños J.E. (2008). Clinical Characteristics and Psychopathological Profile of Patients with Vulvodynia: An Observational and Descriptive Study. Dermatology.

[21] Forth H.L., Cramp M.C., Drechsler W.I. (2009). Does Physiotherapy Treatment Improve the Self-Reported Pain Levels and Quality of Life of Women with Vulvodynia? A Pilot Study. J. Obstet. Gynaecol..

[22] Desrochers G., Bergeron S., Khalifé S., Dupuis M.J., Jodoin M. (2009). Fear Avoidance and Self-Efficacy in Relation to Pain and Sexual Impairment in Women with Provoked Vestibulodynia. Clin. J. Pain..

[23] Ponte M., Klemperer E., Sahay A., Chren M.-M. (2009). Effects of Vulvodynia on Quality of Life. J. Am. Acad. Dermatol..

[24] Xie Y., Shi L., Xiong X., Wu E., Veasley C., Dade C. (2012). Economic Burden and Quality of Life of Vulvodynia in the United States. Curr. Med. Res. Opin..

[25] Lamvu G., Nguyen R.H.N., Burrows L.J., Rapkin A., Witzeman K., Marvel R., Hutchins D., Witkin S., Veasley C., Fillingim R. (2015). The Evidence-Based Vulvodynia Assessment Project: A National Registry for the Study of Vulvodynia. J. Reprod. Med..

[26] Rosen N.O., Bois K., Mayrand M.H., Vannier S.A., Bergeron S. (2016). Observed and Perceived Disclosure and Empathy Are Associated with Better Relationship Adjustment and Quality of Life in Couples Coping with Vulvodynia. Arch. Sex. Behav..

[27] Lamvu G., Alappattu M., Witzeman K., Bishop M., Robinson M., Rapkin A. (2018). Patterns in Vulvodynia Treatments and 6-Month Outcomes for Women Enrolled in the National Vulvodynia Registry—An Exploratory Prospective Study. J. Sex. Med..

[28] Tribó M.J., Canal C., Baños J.E., Robleda G. (2020). Pain, Anxiety, Depression, and Quality of Life in Patients with Vulvodynia. Dermatology.

[29] Patla G., Apatla M., Sharma V., Singh R., Singh K. (2023). Chronic Vulvar Pain and Health-Related Quality of Life in Women with Vulvodynia. Life.

[30] Nimbi F.M., Renzi A., Mesce M., Limoncin E., Galli F. (2024). Central Sensitization Symptoms in Vulvodynia: Exploring the Role of Temperament, Personality Traits, Childhood Adverse Events, Defense Mechanisms, and Mental Pain on Quality of Life. J. Sex. Med..

[31] Çankaya S., Mangır Meler K. (2025). Genitourinary Pain, Sexual Distress, and Quality of Sexual Life in Women with and without Vulvodynia. Sex. Res. Soc. Policy.

[32] Gattamelata A., Fioravanti G., Zurkirch V.P., Moyano N. (2025). Psychological, Relational, and Fertility-Related Characteristics of Italian Women with Vulvodynia: A Comparative Study with Controls. Int. J. Environ. Res. Public Health.

[33] Brotto L.A., Basson R., Carlson M., Zhu C. (2013). Impact of an Integrated Mindfulness and Cognitive Behavioural Treatment for Provoked Vestibulodynia (IMPROVED): A Qualitative Study. Sex. Relatsh. Ther..

[34] Groven K.S., Råheim M., Håkonsen E., Haugstad G.K. (2015). “Will I Ever Be a True Woman?” An Exploration of the Experiences of Women with Vestibulodynia. Health Care Women Int..

[35] LePage K., Selk A. (2016). What Do Patients Want? A Needs Assessment of Vulvodynia Patients Attending a Vulvar Diseases Clinic. Sex. Med..

[36] Montali L., Bernareggi C., Crispiatico V. (2025). Aggravating and Protective Factors in Patients’ Experiences of Vulvodynia: A Qualitative Study with Italian Women. BMC Psychol..

[37] Harryson J., Sjöström R. (2025). Provoked Vulvodynia from a Patient Perspective—Physiotherapy Made a Difference. Eur. J. Physiother..

[38] Rosen N.O., Dawson S.J., Brooks M., Kellogg-Spadt S. (2019). Treatment of vulvodynia: Pharmacological and non-pharmacological approaches. Drugs.

[39] Andrews J.C. (2010). Vulvodynia: An evidence-based approach to medical management. Curr. Obstet. Gynecol. Rep..

[40] Falsetta M.L., Foster D.C., Bonham A.D., Phipps R.P. (2017). A review of the available clinical therapies for vulvodynia management and new data implicating pro-inflammatory mediators in pain elicitation. BJOG Int. J. Obstet. Gynaecol..

[41] Calafiore D., Marotta N., Curci C., Agostini F., De Socio R.I., Inzitari M.T., Ferraro F., Bernetti A., Ammendolia A., de Sire A. (2024). Efficacy of rehabilitative techniques on pain relief in patients with vulvodynia: A systematic review and meta-analysis. Phys. Ther. Rehabil. J..

