# Supplementary material for: Impact of Vulvodynia on the Quality of Life of Women: A Rapid Review
Source: J Clin Med. 2025 Dec 22;15(1):70. doi: 10.3390/jcm15010070 (PMC12787231; doi:10.3390/jcm15010070)
Supplement: Supplementary file 1 [file jcm-15-00070-s001.zip › jcm-4025585-supplementary.pdf]

Suplementary material S1

Databases consulted and search processing

**Table 1: summary of databases consulted and documents retrieved**		
**Database**	**Search strategy**	**Nº of documens**
Pubmed	("Vulvodynia"[Mesh]) AND "Quality of Life"[Mesh]	63
Web of Science (WoS)	("Vulvodynia" OR "Vulvodinya" OR "Vestibulodynia" OR "Generalized Vulvodynia" OR "Vulvodynia, Generalized" OR "Vulva Pain" OR "Pain, Vulva") (Title) and ("quality of life" OR "Life Quality" OR "Health-Related Quality Of Life" OR "Health Related Quality Of Life" OR "HRQOL") (All Fields) and Article (Document Types)	64
Scopus	TITLE ("Vulvodynia" OR "Vulvodinya" OR "Vestibulodynia" OR "Generalized Vulvodynia" OR "Vulvodynia, Generalized" OR "Vulva Pain" OR "Pain, Vulva") AND TITLE-ABS-KEY ("quality of life" OR "Life Quality" OR "Health-Related Quality Of Life" OR "Health Related Quality Of Life" OR "HRQOL") AND (LIMIT-TO (DOCTYPE , "ar"))	100
Biblioteca virtual de salud	(ti:((vulvodinia OR vulvodynia OR vulvodinya OR vestibulodynia OR "dolor vulvar" OR "vulva pain" OR "pain vulva"))) AND (("quality of life" OR "Life Quality" OR "Health-Related Quality Of Life" OR "Health Related Quality Of Life" OR "HRQOL"))	10
Cuiden	(vulvodinia OR "dolor vulvar")	0
CINHAL	TI ((vulvodynia OR vulvodinya OR vestibulodynia OR "generalized vulvodynia" OR "vulvodynia, generalized" OR "vulva pain" OR "pain, vulva")) AND (("quality of life" OR "Life Quality" OR "Health-Related Quality Of Life" OR "Health Related Quality Of Life" OR "HRQOL"))	49

Suplementary material S2.

Checklist for selection criteria of the articles

Author and year	S1. Are there clear research questions?	S2. Do the collected data allow to address the research questions?	4.1. Is the sampling strategy relevant to address the research question?	4.2. Is the sample representative of the target population?	4.3. Are the measurements appropriate?	4.4. Is the risk of nonresponse bias low?	4.5. Is the statistical analysis appropriate to answer the research question?	Overall assessment/ risk of bias (MMAT)	Main limitations
**Arnold et al., 2006 [17]**	Yes	Yes	Partial	Yes	Partial	Yes	Yes	High-moderate	Healthcare sample, possible recall bias, self-reporting without clinical verification of comorbidities.
**Arnold et al., 2007 [18]**	Yes	Yes	Yes	Yes	Partial	Yes	Yes	High	Self-reported diagnosis, lack of clinical validation, exclusion of non-English speakers, potential recall bias
**Tribó et al., 2008 [19]**	Yes	Yes	Partial	Yes	Yes	Yes	Yes	High	Non-probability sampling, clinical sample
**Forth et al., 2009 [20]**	Yes	Yes	Partial	Yes	Yes	Partial	Yes	High-moderate	Small sample, non-random, no parallel control group, short follow-up period, no adjustment for confounders
**Desrochers et al., 2009 [21]**	Yes	Yes	Partial	Yes	Yes	Yes	Yes	High	Cross-sectional design, self-reporting, non-probability sample, no control group, possible reverse causality bias.
**Ponte et al., 2009 [22]**	Yes	Yes	Partial	Yes	Yes	Can´t tell	Yes	High-moderate	Non-representative clinical sample.

									No response rate reported.
**Xie et al., 2012 [23]**	Yes	Yes	Can´t tell	Yes	Partial	Partial	Yes	Moderate	Self-reported diagnosis, non-probability sample, overrepresentation of Caucasian women with higher education, economic self-reporting.
**Lamvu et al., 2015 [24]**	Yes	Yes	Partial	Yes	Yes	Partial	Yes	High-moderate	Unrepresentative clinical sample, loss to follow-up > 40%, and absence of external control group.
**Rosen et al., 2016 [25]**	Yes	Yes	Can´t tell	Yes	Yes	Yes	Yes	High	Small and clinical sample; cross-sectional design; possible social desirability bias; greater representation of young heterosexual couples.
**Lamvu et al., 2018 [26]**	Yes	Yes	Partial	Yes	Partial	Partial	Yes	Moderate	High variability of treatments, no confounding control, loss to follow-up, clinical sample.
**Tribó et al., 2019 [27]**	Yes	Yes	Partial	Yes	Yes	Can´t tell	Yes	High-moderate	Single-center sample, no control group, predominance of women over 40 years of age. Cross-sectional design.
**Patla et al., 2023 [28]**	Yes	Yes	Partial	Yes	Partial	Can´t tell	Yes	Moderate	Small sample size, non-random online

									recruitment, lack of control group, and subjective self-reporting.
**Nimbi et al., 2024 [29]**	Yes	Yes	Partial	Yes	Partial	Yes	Yes	High-moderate	Self-reported diagnosis and responses; exclusively Italian sample; cross-sectional design (no causality).
**Çankaya & Meler, 2025 [30]**	Yes	Yes	Partial	Yes	Yes	Yes	Yes	High	Local sample, convenience recruitment, self-reporting, and lack of external validation.
**Gattamelata et al., 2025 [31]**	Yes	Yes	Partial	Yes	Yes	Yes	Yes	High	Self-selected sample, cross-sectional design, self-report, and absence of some clinical variables

Table 3.Risk of bias evaluation — Qualitative studies (n = 7)

Main author and year	S1. Are there clear research questions?	S2. Do the collected data allow to address the research questions?	1.1. Is the qualitative approach appropriate to answer the research question?	1.2. Are the qualitative data collection methods adequate to address the research question?	1.3. Are the findings adequately derived from the data?	1.4. Is the interpretation of results sufficiently substantiated by data?	1.5. Is there coherence between qualitative data sources, collection, analysis and interpretation?	Overall assessment (MMAT)	Main limitations
**Brotto et al., 2013 [32]**	Yes	Yes	Yes	Yes	Yes	Yes	Yes	High	Small, clinical sample; possible memory bias.
**Groven et al., 2015 [33]**	Yes	Yes	Yes	Yes	Yes	Yes	Yes	High	Small, homogeneous sample; possible influence of interviewers.
**LePage & Selk, 2016 [34]**	Yes	Yes	Yes	Yes	Yes	Yes	Yes	High	Small, single-center sample.
**Montali et al., 2025 [35]**	Yes	Yes	Yes	Yes	Yes	Yes	Yes	High	Self-selected sample, interviews in a specific cultural context.
**Harryson & Sjöström, 2025 [36]**	Yes	Yes	Yes	Yes	Yes	Yes	Yes	High	Small sample, digital interviews, possible researcher bias, limited cultural context.
